# Historical development of accelerometry measures and methods for physical activity and sedentary behavior research worldwide: A scoping review of observational studies of adults

**DOI:** 10.1371/journal.pone.0276890

**Published:** 2022-11-21

**Authors:** Kelly R. Evenson, Elissa Scherer, Kennedy M. Peter, Carmen C. Cuthbertson, Stephanie Eckman

**Affiliations:** 1 Department of Epidemiology, Gillings School of Global Public Health, University of North Carolina–Chapel Hill, Chapel Hill, North Carolina, United States of America; 2 RTI International, Research Triangle Park, North Carolina, United States of America; Universiti Malaya, MALAYSIA

## Abstract

This scoping review identified observational studies of adults that utilized accelerometry to assess physical activity and sedentary behavior. Key elements on accelerometry data collection were abstracted to describe current practices and completeness of reporting. We searched three databases (PubMed, Web of Science, and SPORTDiscus) on June 1, 2021 for articles published up to that date. We included studies of non-institutionalized adults with an analytic sample size of at least 500. The search returned 5686 unique records. After reviewing 1027 full-text publications, we identified and abstracted accelerometry characteristics on 155 unique observational studies (154 cross-sectional/cohort studies and 1 case control study). The countries with the highest number of studies included the United States, the United Kingdom, and Japan. Fewer studies were identified from the continent of Africa. Five of these studies were distributed donor studies, where participants connected their devices to an application and voluntarily shared data with researchers. Data collection occurred between 1999 to 2019. Most studies used one accelerometer (94.2%), but 8 studies (5.2%) used 2 accelerometers and 1 study (0.6%) used 4 accelerometers. Accelerometers were more commonly worn on the hip (48.4%) as compared to the wrist (22.3%), thigh (5.4%), other locations (14.9%), or not reported (9.0%). Overall, 12.7% of the accelerometers collected raw accelerations and 44.6% were worn for 24 hours/day throughout the collection period. The review identified 155 observational studies of adults that collected accelerometry, utilizing a wide range of accelerometer data processing methods. Researchers inconsistently reported key aspects of the process from collection to analysis, which needs addressing to support accurate comparisons across studies.

## Introduction

“Accelerometry” refers to device-based motion sensors that provide detailed movement information by capturing changes in a person’s gravitational acceleration in space [1]. The first accelerometers, developed in the 1920’s, weighed about one pound and measured the vibration of aircraft and large structures such as bridges [2]. In the 1950’s, accelerometers measured gait velocity [3], and by the 1970’s their potential for measurement of human movement was recognized [4]. Researchers began adopting accelerometry as an indicator of physical activity in the 1980’s, and Troiano et al. [5] estimated that they have been used in epidemiologic studies for research and surveillance since the mid-1990’s. A number of technologic advances in accelerometry sensors occurred since the 1990’s, including increased storage, longer battery life, wider acceleration range, waterproofing, and smaller size [1, 5]. These technologic advances, along with the relative improvement in cost and validity over time of the device to represent physical activity and sedentary behavior, contributed to subsequent rise in the application of the device by researchers.

The use of accelerometry to measure physical activity and sedentary behavior (together referred to as “physical behavior”) was a significant milestone in the field. Accelerometers enabled measurement of detailed components of the behaviors in conditions where self-reports were not possible (i.e., young age, cognitive impairment, assessment of light physical activity, or bouts of physical activity) and allowed for both cross-language and cross-population comparisons. Entire networks, such as the International Physical Activity and the Environment Network (IPEN; https://www.ipenproject.org/) and the Prospective Physical Activity, Sitting, and Sleep consortium (ProPASS; https://www.propassconsortium.org/), facilitate harmonization of accelerometer methods and data analysis between global research endeavors. However, not enough effort has been dedicated to fully understanding where and how physical behavior research using accelerometers is happening. Systematically cataloging these studies and their reporting methods has the potential to increase global collaborations and harmonization efforts.

While the use of accelerometry expanded over the last four decades, reporting in scientific studies on key aspects of the devices and decisions made in processing the data remains inconsistent. Calls for improvement in reporting date back to at least 2004, at an international meeting focused on accelerometry measurement for physical activity. The conference recommended that researchers state their decision rules for collecting, processing, and analyzing the data, and that they work towards developing common practices and guidance [6, 7]. More complete reporting of accelerometry methods is necessary in order to compare across studies, and promote standardized decision rules to facilitate future harmonization with studies using these devices, such as in meta analyses [8–10]. Several seminal papers on best practices using accelerometry in population-based research provide guidance on key accelerometry information to report, such as the number of participants enrolled and accelerometry wear and nonwear definitions [11, 12]. It is not known how well studies follow these accelerometry reporting guidelines.

To address these issues, we conducted a scoping review to identify and describe observational studies that utilized accelerometry to assess physical activity and sedentary behavior. From the studies found, we abstracted key study information and then applied an accelerometry reporting tool to describe the completeness of describing key information [13]. This work was done in order to describe the current state of the science and reporting practices for accelerometry, and to identify and facilitate future global collaborations and harmonization across studies.

## Materials and methods

### Search methods

The systematic review protocol was developed in accordance with the Preferred Reporting Items for Systematic Review and Meta-Analysis Protocols (PRISMA-P) statement [14]. The PRISMA Scoping Review checklist [15] can be found in **S1 File**. Since this review focused on accelerometry measurement and was a scoping rather than systematic review [16], it did not fit the current criteria to register with PROSPERO [17]. We searched three databases (PubMed, Web of Science, and SPORTDiscus) on June 1, 2021 for articles published up to that date, with the search strategy detailed in **S2 File**. After removing duplicate citations, two authors independently screened all titles/abstracts and full-text articles for inclusion using Covidence systematic review software (www.covidence.org; Veritas Health Innovation, Melbourne, Australia) with discrepancies resolved by consensus.

### Inclusion and exclusion criteria

Inclusion criteria included observational studies, including surveillance studies, with analytical sample sizes of at least 500 community-dwelling adults 18 years and older who wore an accelerometer for the purposes of collecting physical activity and sedentary behavior. We included studies that used accelerometry to collect physical activity (including steps) or sedentary behavior, regardless of whether raw or proprietary-based metrics were used. Studies needed to be described in full-length peer-reviewed papers in English. If there was more than one publication identifying a single study that met our criteria for inclusion, then we included one publication to represent the study, using the study that provided the most information we were abstracting. If needed, we sought missing information from other publications captured by the search.

In cases in which a protocol was referenced in the main paper, information was also abstracted from the protocol. We reviewed all publications identified for each study to determine the best source paper. For example, we identified 29 papers that published on the United Kingdom Biobank Study, but only one was included in the sample. Several publications identified in our search included data from multiple studies in a single publication; as long as the unique study met inclusion criteria, they were retained. Surveillance studies that recruited a unique set of participants for each wave were included as separate studies. For example, NHANES 2003–2004 [18] and NHANES 2005–2006 [19] were counted as separate studies. For cohort studies with multiple waves of accelerometer data collection, we did not count multiple waves as separate studies.

**Fig 1:**
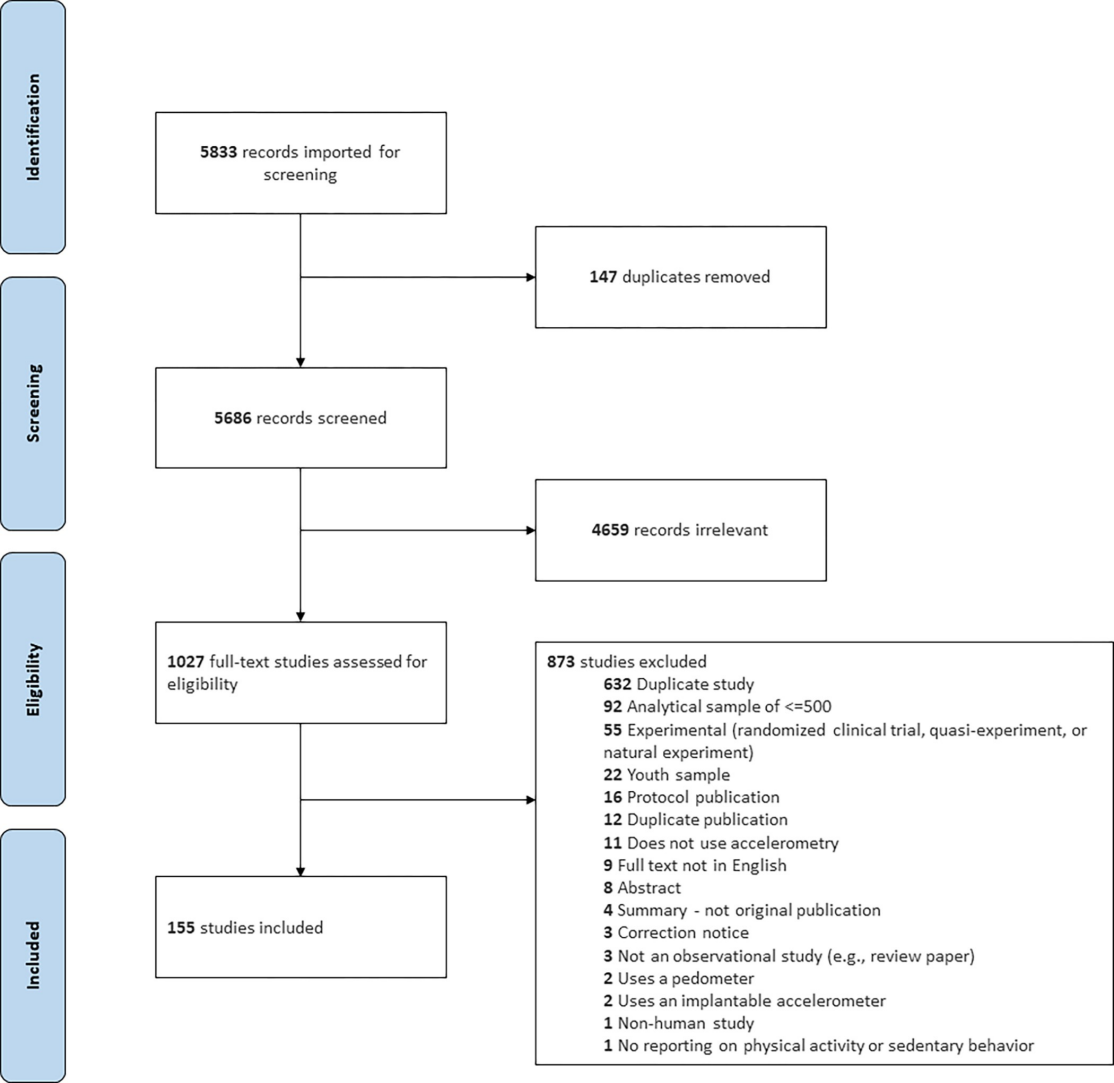
PRISMA chart displaying the identification, screening, eligibility and inclusion for each study reviewed. In total, 155 studies were included from 154 publications. One publication included two studies (Dutch Longitudinal Internet Studies for Social Sciences and Understanding America Study) that were not mentioned in other identified publications.

We excluded publications in the grey literature, abstracts, dissertations, and conference proceedings. We excluded studies of hospitalized or institutionalized adults, or samples that gave consent by proxy, as well as studies of youth (children or adolescents <18 years of age). We excluded intervention studies (i.e., randomized trials, quasi-experimental trials), unless there was a new consent process that enrolled participants into an observational study. We excluded studies that used spring-levered pedometers, but included pedometers that used accelerometry, as further distinguished elsewhere [20]. Studies that collected accelerometry, but did not report on physical behaviors, were excluded. For example, Scarlett et al. [21] collected accelerometry data but only used it to describe sleep, and was therefore excluded. If a publication met multiple exclusion criteria, it was excluded in the priority order in which they appear in **Fig 1**.

### Abstraction

Using Covidence software, one rater abstracted the information and a second rater checked the abstraction, with discrepancies resolved by consensus. The abstraction tool included the following domains: study information (e.g., study design, gender, country, years of accelerometry collection, population-based sampling, sample weights used, logbook kept, number of accelerometers worn, analytic sample size), brand and settings (e.g., sampling frequency, epoch length), method of distribution, and method of return. If years of accelerometry collection was missing, we made an attempt to contact the authors for the information. We counted a study as using population-based sampling if the sampling frame was clearly defined and allowed for inference to an underlying reference population. If the geography that the sample came from was well defined, regardless of how small it was, we counted it. Sampling from schools or clinics was not included unless all schools or clinics from a defined geographic area were part of the sampling frame. We identified a data collection protocol as being a “distributed donor” if participants connected their own commercial devices that contained an accelerometer (e.g., Fitbit) to an application to share their data with researchers.

We also collected accelerometer wear (e.g., days of data collection, days required, wear protocol, diary used), nonwear (e.g., criteria to define nonwear, adherent days of wear for analysis, adherent time of wear for analysis, weekend wear), placement, and attachment. For nonwear algorithms, when the Choi algorithm was referenced [22], we assumed that nonwear was defined as > = 90 consecutive minutes of zero counts, with a movement window of up to 2 minutes and an upstream and downstream window of 30 minutes. Similarly, when the NHANES algorithm was referenced [23], we assumed that nonwear was defined by an interval of > = 60 consecutive minutes of zero counts with a movement window up to 2 minutes between 0–100 counts/minute. When abstracting analytic sample size, in cases where multiple accelerometers were worn, we reported on the largest sample size for one accelerometer.

A random sample of 49 studies was selected for further contact using two attempts with at least two different people associated with the study. Studies were selected with probability proportional to the number of publications identified from the search that belonged to the study and stratified by region. For these 49 studies, an intake sheet was created with abstracted information about accelerometry from all publications and the study website. In total, 26 responded by reviewing, correcting, or completing missing accelerometry information.

When assessing the completeness of reporting, we applied the accelerometry reporting tool developed by Montoye et al. [13] to the data we extracted from the published papers. Specifically for the scoring of the tool, we did not include supplemental information obtained through study contact with 26 studies. The reporting tool included 12 items: 7 questions on accelerometer information, 4 questions on data processing and interpretation, and 1 question on protocol non-compliance which we modified for our purposes (**S3 File**).

### Analysis

Analyses were conducted by study (n = 155) and by accelerometer (n = 166), as some studies had participants wear multiple accelerometers, and sometimes multiple brands. These two datasets are publicly accessible elsewhere [24]. In this review, percentages were reported for categorical variables and means with standard deviations were reported for continuous variables. These analyses were conducted in SAS (Cary, North Carolina). We created a map to indicate location using the rworldmap package in R [25, 26].

## Results

### Study selection

A total of 5686 records were screened with 1027 full-text studies assessed for eligibility. Among those, 873 were excluded, resulting in a final list of 155 unique observational studies of adults using accelerometry to measure physical activity or sedentary behavior (Fig 1) [27–175]. Four publications referred to more than one included study [52, 53, 110, 164]. Most of the 873 studies were excluded because they represented a publication from a study we already included (n = 632), but other common reasons included an analytic sample size of less than 500 (n = 92), experimental study design (n = 53), or a youth sample less than 18 years without at least 500 adults (n = 22). Other reasons can be found in Fig 1.

### Study description

From 155 included observational studies, all were cross-sectional or cohort except for 1 case control study. Five (3.2%) studies, all published in 2020–2021, utilized a distributed donor protocol, whereby participants in the study remotely connected their personal device with an accelerometer to an application to share accelerometer data with researchers (**Table 1**). Overall, 8.4% of the studies enrolled females only, while 2.6% enrolled males only.

**Table 1:** Description of observational studies of adults collecting accelerometry (n = 155).

	Overall	
**Description**	**%**	**n**
**Starting year of data collection:**		
1999–2004	5.2	8
2005–2009	30.3	47
2010–2014	40.0	62
2015–2021	24.5	38
**Distributed donor protocol:**		
Yes	3.2	5
No	96.8	150
**Gender:**		
Male	2.6	4
Female	8.4	13
Male and Female	89.0	138
**Population based:**		
Yes	51.0	79
No or not indicated	49.0	76
**Sample weights used:**		
Yes	12.9	20
No or not indicated	87.1	135
Analytic sample size*:		
500–700	24.5	38
701–1000	22.6	35
1001–2250	27.1	42
> 2250	25.8	40
**Log book kept:**		
Yes	29.7	46
No or not indicated	70.3	109
**Number of accelerometers worn:**		
1	94.2	146
2	5.2	8
3	0.0	0
4	0.6	1

*In a few cases, the analytic sample size may include youths less than 18 years of age.

**Fig 2:**
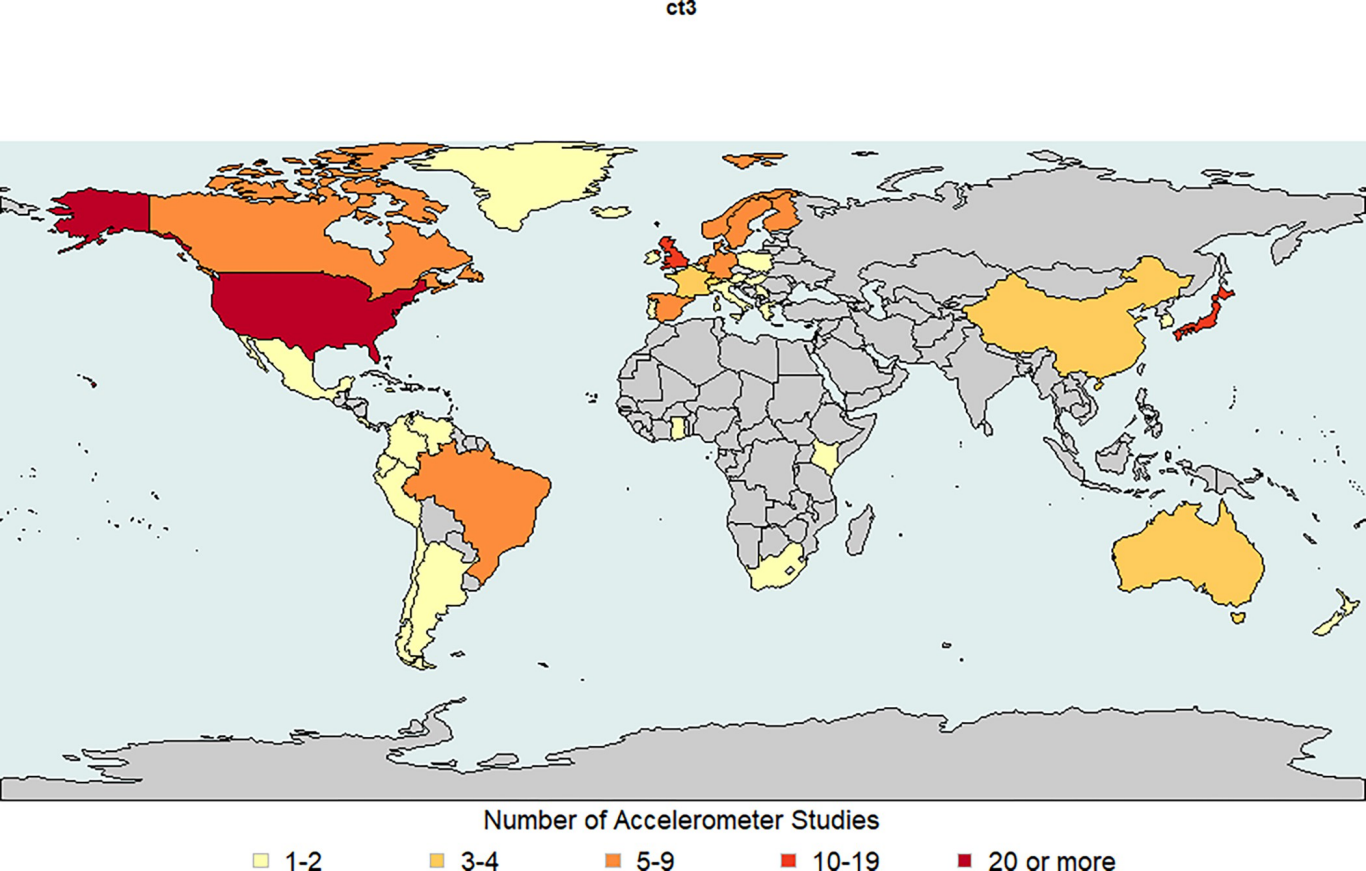
World map displaying the frequency of accelerometry studies found by country (n = 150). This map does not include the 5 distributed donor studies. England, Scotland, Wales, and Northern Ireland were mapped as the United Kingdom. Republished from [26] under a CC BY license, with permission from Dr. Andy South, original copyright 2011.

Accelerometry collection occurred in 31 countries, in addition to 7 studies that collected in more than one country (S4 File). Fig 2 displays the country where participants lived, revealing a high number of studies in Japan (n = 16), the United Kingdom (including England, Scotland, Wales, and Northern Ireland) (n = 16), and the United States (n = 43). The continents of Africa, Asia (with the exception of Japan), and Australia had much lower representation.

**Fig 3:**
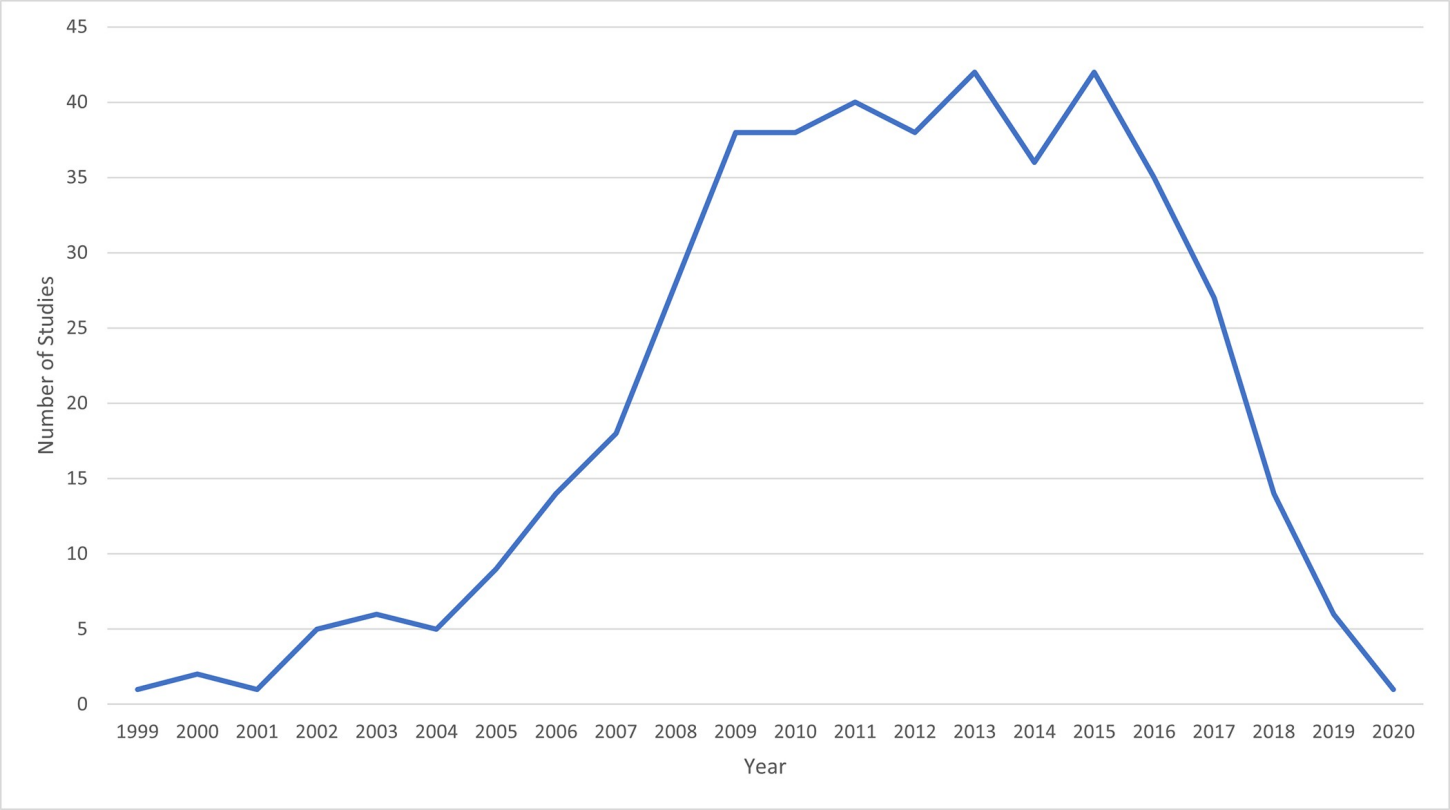
Number of studies collecting data by year (n = 155). Note that if a study collects accelerometry over multiple years, then it is included on the graph in each of those years.

The earliest study year with accelerometry data collection was 1999, [175] with a noticeable rise in usage from 2004 to 2009 (Fig 3). The declining data collection in 2018 to 2020 is attributable to the time it takes to collect, process, analyze, and publish results in relation to our search date.

Approximately half (51.0%) of the studies used population-based sampling (**Table 1**). However, only 12.9% used sample weights. Two studies did not meet our definition of population-based, but did create sampling weights for their study [63, 143]. In terms of sampling, the majority of studies enrolled community dwelling adults, but 13 studies selected participants based on the following health conditions: first bariatric surgery (n = 1) [114], females experiencing infertility (n = 1) [128], fibromyalgia (n = 1) [27], survivors of acute lymphoblastic leukemia (n = 1) [97], and pregnancy (n = 2) [54, 134], and postmenopause (n = 1) [120]. In addition, several studies selected participants with or at risk for knee osteoarthritis (n = 2) [63, 172], and diabetes or specific blood glucose levels (n = 4) [40, 86, 89, 117]. Analytic sample sizes ranged from 512 to 8,203,261, with a median of 1095 (interquartile range 703 to 2325) and mean 55,580.

Almost one-third (29.7%) of studies asked participants to keep a logbook of accelerometer wear time, nonwear time, and/or sleep time (**Table 1**). A few studies also specified a protocol to capture workday activities (n = 5) [84, 107, 108, 119, 162], bicycling (n = 3) [63, 110], swimming/water activities (n = 2) [32, 63], and outdoor activities (n = 1) [141]. Most studies required wear of one accelerometer (94.2%); however, 8 studies (5.2%) asked participants to wear two accelerometers at the same time and 1 study (0.6%) asked participants to wear four accelerometers at the same time.

### Accelerometer characteristics

Table 2 displays the accelerometer characteristics based on the total number of accelerometers worn [147 studies with one accelerometer] + (8*2) [8 studies with 2 accelerometers] + (1*4) [one study with 4 accelerometers] = 166). Overall, studies used 25 different brands of accelerometers, with the most popular including the ActiGraph (46.4%), Actical (8.4%), and GENEACtiv (7.8%). Most studies did not report on sampling frequency, but among those that did the most common setting was 30 Hz, ranging from 5 Hz to 100 Hz. The epoch length ranged from 1 second to 5 minutes, with 21 accelerometers capturing raw data that was used in the publication.

**Table 2:** Description of accelerometers used by observational studies of adults (n = 166).

	Overall	
**Description**	**%**	**n**
**Accelerometer Brand and Settings**	** **	** **
**Brand of accelerometer:**		
Actiband	0.6	1
Actibelt	0.6	1
Actical	8.4	14
ActiGraph (includes Computer Science Application, Inc.)	46.4	77
ActiHeart	4.8	8
ActivPAL	3.6	6
Actiwatch Spectrum	1.8	3
Ambulator	0.6	1
Axivity	1.8	3
Bong II	0.6	1
Fitbit	4.2	7
GENEActiv	7.8	13
Hookie	1.8	3
Kao	0.6	1
Lifecorder	3.0	5
Omron Active style Pro	4.2	7
Orthocare Stepwatch	1.2	2
Panasonic Actimarker	0.6	1
Polar	1.2	2
RT3	0.6	1
SenseWear	2.4	4
StepWatch	0.6	1
UKK	1.2	2
Withings	0.6	1
X15-1c	0.6	1
**Sampling frequency in Hz:**		
5	2.4	4
20	1.8	3
30	15.7	26
32	6.6	11
50	3.0	5
60	1.8	3
80	0.6	1
85.7	3.0	5
100	6.7	11
Not indicated or not a choice	58.4	97
**Epoch length:**		
1 second	5.4	9
4 seconds	1.2	2
5 seconds	3.6	6
6 seconds	0.6	1
10 seconds	3.6	6
15 seconds	3.0	5
30 seconds	6.6	11
1 minute	41.6	69
2 minutes	0.6	1
5 minutes	0.6	1
Raw and raw with epochs of 1 to 60 seconds	12.7	21
Not indicated or not a choice	20.5	34
**Accelerometer Distribution and Return:**		
**Method of accelerometer distribution:**		
In-person	43.4	72
Mail	17.5	29
Mailed or in-person	1.8	3
Not applicable (e.g., distributed cohort)	3.0	5
Not indicated	34.3	57
**Method of accelerometer return:**		
In-person	16.9	28
Mail	30.7	51
Device not asked to be returned	0.6	1
Not applicable (e.g., distributed cohort)	3.0	5
Not indicated	48.8	81
**Accelerometer Wear**		
**Days of data collection:**		
1 day	0.6	1
2 days	1.2	2
3 days	1.2	2
4 days	4.2	7
5 days	2.4	4
6 days	1.2	2
7 days	62.0	103
4 to 7 days variable by participant	1.8	3
8 days	6.6	11
9 to 14 day range	9.6	16
Other	5.4	9
Not indicated	3.6	6
**Wear protocol:**		
Wake only	45.2	75
24 hours (including sleep)	44.6	74
Not indicated	10.2	17
**Accelerometer Non-wear**		
Weekend wear required to be adherent:		
Yes	11.4	19
No/Not indicated	88.6	147
Number of adherent days of wear to be used in the analysis:		
1 day	6.6	11
2 days	5.4	9
3 days	13.9	23
4 days	38.0	63
5 days	9.6	16
6 days	0.6	1
7 days	5.4	9
Other	3.6	6
Not indicated	16.9	28
Number of minutes/day of wear to be an adherent day:		
480 minutes (8 hours)	4.2	7
600 minutes (10 hours)	59.0	98
800 minutes (12 hours)	0.6	1
840 minutes (14 hours)	0.6	1
960 minutes (16 hours)	3.0	5
1080 minutes (18 hours)	0.6	1
1200 minutes (20 hours)	1.2	2
1440 minutes (24 hours)	2.4	4
Other	8.4	14
Not indicated	19.9	33

Note: Eight studies used 2 accelerometers, and one study used 4 accelerometers; therefore, the sample size was n = 166 for this table. We selected a sample of studies and n = 29 responded to check their entries and fill in missing information when possible. Therefore, the "not indicated" category is reduced when the study provided missing information from the selected study.

**Fig 4:**
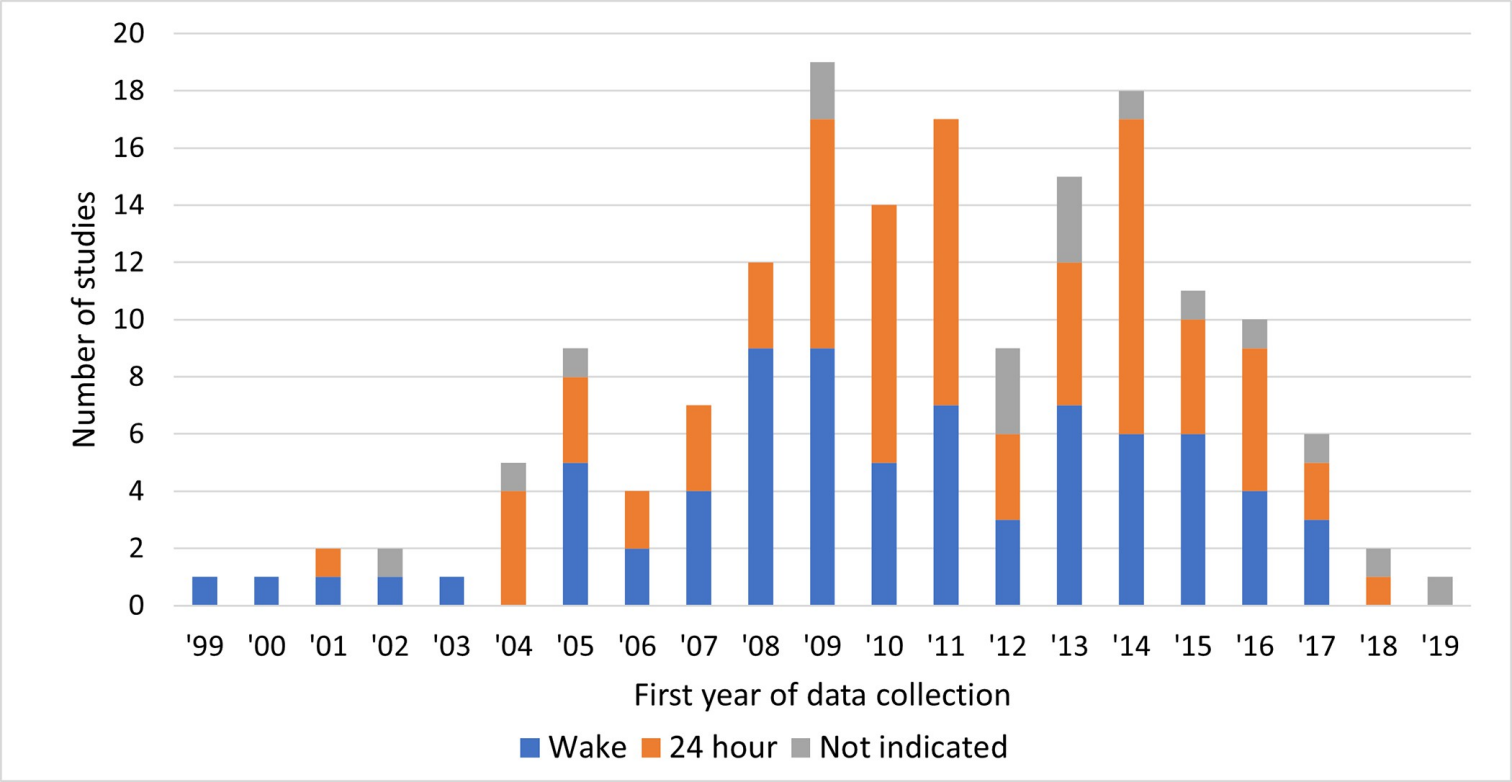
Wear protocol by first year of data collection, graphed at the accelerometer level (n = 166).

Accelerometer distribution was more frequently in-person (n = 71) rather than mailed (n = 29) (Table 2). In contrast, mail (n = 51) was the more common return method over in-person (n = 28). The most common number of days of data collection was 7 days (n = 103), although 32 accelerometers collected 8 or more days of wear. Four accelerometer protocols specified weekend days of wear. For the accelerometry wear protocols, 75 required wake only and 74 required continuous wear including sleep. When exploring by the first year of data collection (Fig 4), we found a larger proportion of studies using a 24-hour protocol compared to a wake only protocol starting in 2010 and following for most years.

The studies applied a wide variety of algorithms to remove nonwear time. Most of them were based on consecutive zeros, and many algorithms accounted for a short interruption period (S5 File). For 20 accelerometers, nonwear was identified using raw data. Uniquely, we found a few studies used a capacitive sensor (n = 1) [124], galvanic heat sensor (n = 1) [94], heart rate (n = 1) [160], or a logbook (n = 2) [76, 88] to distinguish wear from nonwear periods. The number of required adherent days (regardless of the physical activity or sedentary behavior metric) ranged from 1 to 7 days, with the most common of 4 days used (n = 64) (Table 2). The number of hours to wear an accelerometer to be defined as an adherent day was most often specified for at least 10 hours/day of wear (n = 98). A few studies described individually calibrating the accelerometer using a step test (n = 4 on the whole sample [50, 86, 160] or a subsample [55]) or a treadmill test (n = 1) [173].

**Fig 5:**
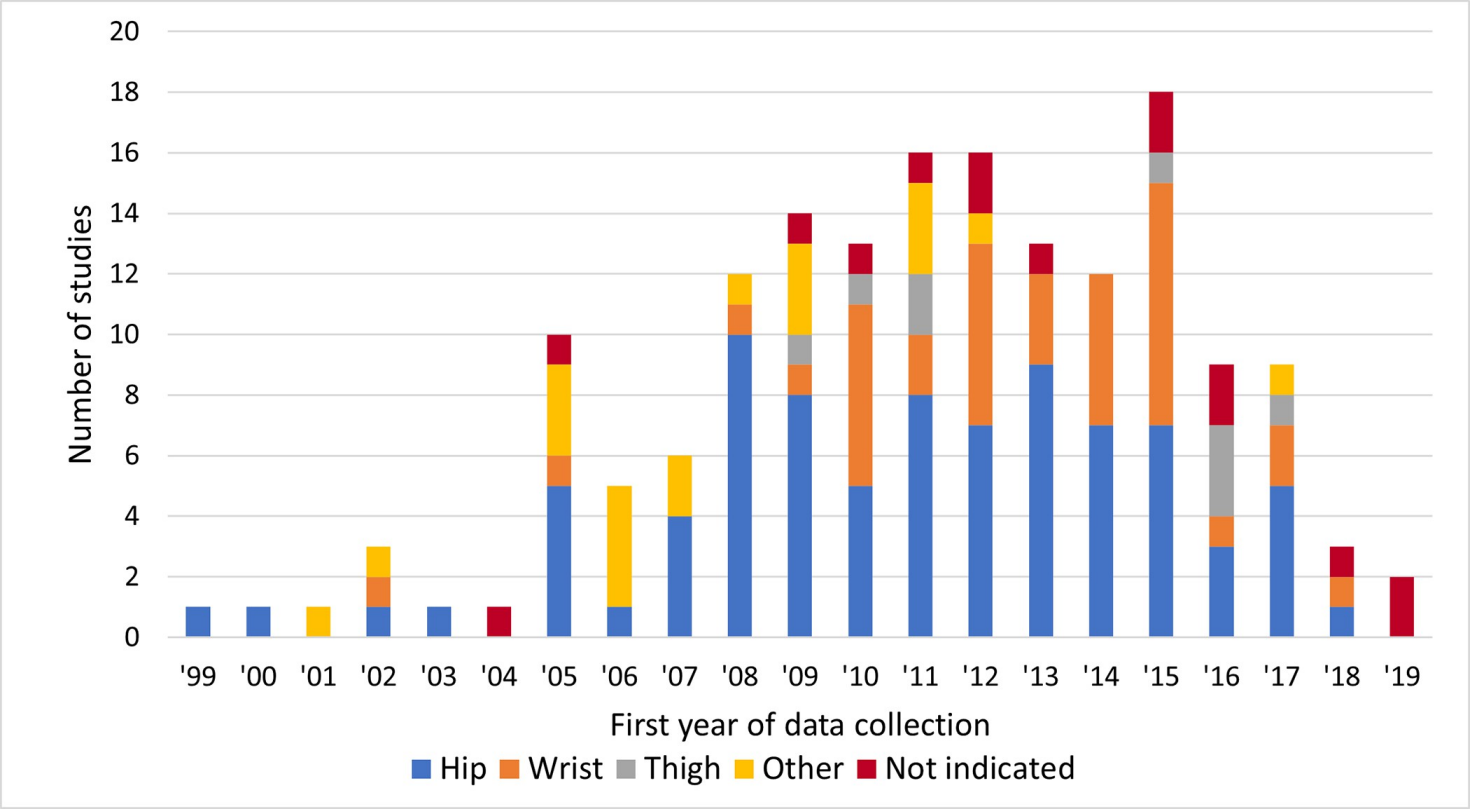
Accelerometer wear location by first year of data collection, graphed at the accelerometer level (n = 166).

The most common accelerometer placement was the hip or waist (n = 82), followed by the wrist (n = 37) and the thigh (n = 9) (Table 3). When exploring by the first year of data collection (Fig 5), hip remained most common by year but wrist collection increased starting around 2010 (with 4 studies collecting wrist data prior to that year). For the hip placement, accelerometer protocols specified wearing it on the right side (n = 47), either side (n = 7), left side (n = 4), dominant side (n = 2), nondominant side (n = 3), on lower back (n = 1), or not indicated (n = 18). For the wrist placement, accelerometer protocols specified nondominant side (n = 29), dominant side (n = 1), nondominant side (n = 1), right side (n = 1), or not indicated (n = 5). For the thigh placement, accelerometer protocols specified right side (n = 5), dominant side (n = 1), or not indicated (n = 3).

**Table 3:** Description of accelerometer placement and attachment method used by observational studies of adults (n = 166).

	Overall	
Description	%	n
**Placement location:**		
Hip/waist	48.4	82
Wrist	22.3	37
Thigh	5.4	9
Chest	4.8	8
Back	3.0	5
Tricep	1.8	3
Ankle	1.2	2
Arm	1.2	2
Hip or lower back	0.6	1
Waist or elsewhere to be clipped on clothing	0.6	1
Wrist or clipped onto belt or clothing	0.6	1
Not indicated	9.0	15
**Placement Side:**		
Right	33.7	56
Left	2.4	4
Either side	4.2	7
Non-dominant	19.9	33
Dominant	3.0	5
Lower back	3.0	5
Upper back	0.6	1
Not indicated	33.1	55
**Attachment method:**		
Adhesive	6.0	10
Adhesive, medical grade	0.6	1
Band	7.8	13
Belt	27.7	46
Clip	1.2	2
Electrode	3.6	6
Strap	0.6	1
Tape	0.6	1
Wristband	4.2	7
Other	1.2	2
Not indicated	46.4	77

Note: Eight studies used 2 accelerometers, and one study used 4 accelerometers; therefore, the sample size was n = 166 for this table. We selected a sample of studies and n = 29 responded to check their entries and fill in missing information when possible. Therefore, the "not indicated" category is reduced when the study provided missing information from the selected study.

### Completeness of reporting accelerometry information

Using 12 items, we evaluated the completeness of reporting on the accelerometer (Table 4). The report is at the accelerometer level (n = 166), rather than the study level (n = 155), since some completeness of reporting differed by accelerometer even within the same publication. Overall, 9.6% reported all 12 items, 15.7% reported 11 items, and 22.4% reported 10 items. The overall mean reporting score was 9.5 (SD 1.9) items reported. The items reported less often included accelerometer return method (41.6%), number of accelerometers distributed (50.0%), and distribution method for sending out accelerometers (57.8%).

**Table 4:** Accelerometry reporting using a tool from Montoye et al. [13] (n = 166).

	Overall	
Reporting	%	n
**Brand of accelerometer used:**		
yes	100	166
**Model of accelerometer used:**		
yes	73.5	122
**Epoch length used:**		
yes	78.3	130
**Accelerometer placement:**		
Yes both location and side	63.9	106
Yes but either location or side only	24.9	43
No	10.2	17
**Sample size of accelerometers distributed:**		
yes	50.0	83
**Accelerometer distribution method out:**		
yes	57.8	96
**Accelerometer distribution method return:**		
yes	41.6	69
**Accelerometer distribution method:**		
Yes out and return	38.0	63
Yes but either out or return only	24.1	40
No	34.9	58
Not applicable	3.0	5
**Days of data collection:**		
yes	96.4	160
**Criteria for non-wear:**		
yes	68.7	114
**Number adherent days:**		
yes	83.7	139
**Time to be considered adherent day:**		
yes	77.1	128
**How meaning was derived:**		
yes	99.4	165
**Sample size not meeting wear time criteria:**		
yes	72.9	121

Note: Eight studies used 2 accelerometers, and one study used 4 accelerometers; therefore, the sample size was n = 166 for this table. Since we collected information from 29 studies about their missing information, the missingness in the prior tables will not match the missingness presented in this table.

The percent indicates that the item was reported, such that the higher the percent the more complete the reporting.

S4 File provides examples of responses to the questions.

## Discussion

Despite widespread use of accelerometry in epidemiological research, a comprehensive list of observational studies leveraging accelerometry to assess physical activity and sedentary behavior did not exist. This scoping review filled this gap by describing the use of accelerometry in 155 observational studies. We documented a growth in the use of accelerometry over time from 1999 to 2019. A marked increase in accelerometry data collection, starting around 2004 to 2009, is congruent with the number of citations in the literature. From 1981 to 1996, fewer than 10 publications per year mentioned physical activity/exercise and accelerometry [176]. This citation index increased to almost 90 per year in 2003–2004 and to more than 600 per year in 2012–2013 [5]. In our review, 20 studies reported collecting accelerometry from more than one time period, offering a glimpse into the prospects of future studies that can potentially account for changes in physical activity and sedentary behavior over time, a limitation of most current studies of accelerometry in association with a health outcome.

We identified 5 studies using distributed donor data, all published since 2020 [65, 131, 153–155]. We expected a rise in this type of study, given the ubiquity of activity trackers in the general population [177], people’s willingness to share their data [178], and the wide-ranging type and amount of granular data collected. It would be important to identify best practices for this study type, given the differences in the way participants might wear the activity tracker without researcher instruction. Researchers should also consider the selectivity in the data collected, because those who own activity trackers tend to be more active, better educated, and younger than those who do not [179]. Among those who own activity trackers, willingness to donate data is related to physical activity and trust in health care providers [178].

Our review identified many areas of the world without accelerometer-based epidemiologic studies, such as large regions on the continents of Africa, Asia, and South America. This finding is consistent with the Global Observatory for Physical Activity Country Cards and Almanac that documented unequal distribution of research productivity by region [180, 181]. These authors point out that in the future, focusing on the global equity of research conducted and the public health impact it makes can contribute to improved physical activity around the world. Lack of physical activity is a worldwide concern, as an estimated 27.5% of adults do not engage in at least 150 minutes/week of moderate intensity, at least 75 minutes/week of vigorous intensity, or an equivalent combination of the two [182]. Moreover, a recent review indicated a decline in physical activity from 1995 to 2017 based on wearable devices collected in 8 countries [183].

Our review collected wear location and attachment method used across studies. As indicated by **Fig 5**, the use of wrist placement is likely increasing due to its greater comfort and lower intrusion, making it easier to wear for a 24-hour protocol. The 24-hour protocol can provide information on sleep, in addition to physical activity and sedentary behavior, to create a 24-hour activity cycle [184]. For wrist placement, the decision to wear the device on the dominant versus the non-dominant hand will impact results, since there is more hand movement on the dominant side that impacts the estimate of sedentary behavior [1, 185, 186]. In our review, most studies that reported on the wrist location used the nondominant side. For hip placement, wearing on the right or left hip may not make much difference [187]. In our review, most studies that reported on the hip location used the right side.

As documented in the 2004 international conference on accelerometry [6], researchers called for device-based companies to provide access to the raw accelerometer signal in order to move away from proprietary-based algorithms that only provided count-based data. Since that time, several accelerometers offered access to the raw signal. This review identified 21 studies that collected raw data. This shift in the field is promising since it facilitates harmonization across device types. Our abstraction tool included a section on machine learning approaches [188], but we did not identify any studies we abstracted using this approach. As data processing improves and algorithms become more widely available, we anticipate that more studies will use the raw signal to identify posture, some types of activities, and finer-grained patterns of physical activity and sedentary behavior.

While approximately half (n = 79) of the studies in the review utilized population-based sampling, only 20 studies used sample weights. When studies do not use these weights, their results may not reflect the characteristics of the population. Stamatakis et al. [189] points out that most observational studies are not representative of the general population due to low response rates. The use of sampling weights helps correct for differential selection probabilities, nonresponse, and other mismatches between the sample and the reference population. When response rates are low, adjustments to the sample weights can be made to reduce the potential for bias due to non-participation, including adjusting for differential nonresponse at the levels of selection and calibration to the census of the underlying geographic area based on sociodemographic characteristics (i.e., age, gender, race/ethnicity). For example, the United Kingdom Biobank Study achieved a 5.5% response rate, and further investigation indicated the presence of bias due to nonresponse [190].

In our review, the completeness of reporting of accelerometer procedures varied across studies. The overall mean reporting score was 9.5, indicating that of 12 key items to report on accelerometry, on average two to three were missing. The completeness of reporting was generally higher than the review that documented reporting from intervention studies [13]. In applying this reporting form, we found in some cases the questions were not relevant for a certain accelerometers, such as specification of epoch length. As more raw data are being used, the reporting tools will need to be updated to reflect these developments.

To our knowledge, this scoping review is the first to systematically identify and describe observational studies of adults with accelerometry measures. However, the scoping review has several limitations that should be acknowledged. First, we generally abstracted one publication per study, so it is possible that missing abstracted fields for a study could be available in other publications. There was a large range of publications for some studies. For example, for the NHANES 2005–2006 wave, the number of publications we collected was 199. Metrics may be differentially reported or more complete in other publications from the same study.

Second, we captured studies with an analytic sample size for accelerometry measurement of at least 500. This designation is somewhat arbitrary and excluded epidemiologic studies with smaller samples. Third, we included only studied published in English, thereby possibly undercounting studies published in other languages. This may also lead to underrepresentation on the map displaying study location. Fourth, although important we did not capture data availability, as this was inconsistently reported particularly in early publications when it often was not a required component of journal articles. Finally, we included studies that used step counting devices that relied on an accelerometer only, and not an older spring-levered configuration. However, we did not specifically search on the term “pedometer”, so studies that focused on step counting using an accelerometer but referred to only as a “pedometer” may have been missed.

## Conclusions

The first peer-reviewed publication in the field of physical activity and public health was published in 1953 by Morris et al. [191] comparing London transport drivers to conductors on incidence of coronary heart disease [192]. The first epidemiologic study of adults (with a sample size of at least 500) that used an accelerometer was published 46 years later, in 1999 [175]. Since 1999, the uptake of the accelerometer into epidemiologic research has been remarkable, as documented by our review.

The database of studies resulting from the review can be useful in identifying potential studies for harmonization and meta analyses using similar protocols and devices [24]. Future efforts could link both health outcomes and data availability to the studies to facilitate research across studies. Our review results indicate the inconsistencies in reporting, supported by prior studies [13, 185], and a wide range of decisions applied to manage and use the data for analysis. The use of a checklist that is completed with journal submission would facilitate more complete reporting. Access to raw accelerometry is becoming more common, and it would benefit the field to create a consensus approach for reporting a set of standardized metrics in order to evaluate key accelerometer decisions, replicate the analysis, and promote harmonization across studies. This review identified regions in the world without any epidemiologic studies of accelerometry.

## Supporting information

S1 File Preferred Reporting Items for Systematic reviews and Meta-Analyses extension for Scoping Reviews (PRISMA-ScR) checklist. (PDF)

S2 File Search strategy used in PubMed, Web of Science, and SPORTDiscus. (PDF)

S3 File Checklist of reporting elements from Montoye et al. [13]. (PDF)

S4 File Location of observational studies of adults collecting accelerometry (n = 155). (PDF)

S5 File Criteria for defining nonwear among accelerometers and combined method of distribution and return used by large observational studies of adults (n = 166). (PDF)
