# Supplementary material for: Historical development of accelerometry measures and methods for physical activity and sedentary behavior research worldwide: A scoping review of observational studies of adults
Source: PLoS One. 2022 Nov 21;17(11):e0276890. doi: 10.1371/journal.pone.0276890 (PMC9678297; doi:10.1371/journal.pone.0276890)
Supplement: S2 File — (PDF) [file pone.0276890.s002.pdf]

Supplement 2. Search strategy used in PubMed, Web of Science, and SPORTDiscus

PubMed

"Motor Activity"[Mesh:NoExp] OR "Exercise"[Mesh] OR "Sedentary Behavior"[Mesh] OR “physical activit*” [Title/Abstract] OR “physical behav*” [Title/Abstract] OR "exercise"[Title/Abstract] OR "sedentary"[Title/Abstract] OR "steps"[Title/Abstract]

AND

"Accelerometry"[Mesh] OR "Fitness Trackers"[Mesh] OR acceleromet*[Title/Abstract] OR fitness track*[Title/Abstract] OR activity monitor*[Title/Abstract] OR motion sensor*[Title/Abstract] OR "device-based"[Title/Abstract] OR "ActiGraph"[Title/Abstract] OR "Actical"[Title/Abstract] OR "Sensewear"[Title/Abstract] OR "Actiband"[Title/Abstract] OR "Actiwatch"[Title/Abstract] OR "FitBit"[Title/Abstract] OR "Garmin"[Title/Abstract] OR "Apple Watch"[Title/Abstract] OR "ActivPAL"[Title/Abstract] OR "Axivity"[Title/Abstract] OR "GeneActiv"[Title/Abstract]

AND

"Cohort Studies"[Mesh:NoExp] OR "Observation"[Mesh] OR "Observational Study"[Publication Type] OR "Observational Studies as Topic"[Mesh] OR cohort*[Title/Abstract] OR "surveillance"[Title/Abstract] OR “cross-sectional” [Title/Abstract] OR observation*[Title/Abstract] OR “Survey” [Title/Abstract]

NOT

"Animals"[Mesh] NOT "Humans"[Mesh] “Randomized Controlled Trial” [Title/Abstract] OR “RCT” [Title/Abstract] OR “intervention” [Title/Abstract]

Total - 4,676 (06/01/2021)

(((((((("Motor Activity"[MeSH Terms:noexp] OR "Exercise"[MeSH Terms]) OR "Sedentary Behavior"[MeSH Terms]) OR "physical activit*"[Title/Abstract]) OR "physical behav*"[Title/Abstract]) OR "Exercise"[Title/Abstract]) OR "sedentary"[Title/Abstract]) OR "steps"[Title/Abstract]) AND ((((((((((((((("Accelerometry"[MeSH Terms] OR "Fitness Trackers"[MeSH Terms]) OR "acceleromet*"[Title/Abstract]) OR "fitness track*"[Title/Abstract]) OR "activity monitor*"[Title/Abstract]) OR "motion sensor*"[Title/Abstract]) OR "device-based"[Title/Abstract]) OR "ActiGraph"[Title/Abstract]) OR "Actical"[Title/Abstract]) OR "Sensewear"[Title/Abstract]) OR "Actiwatch"[Title/Abstract]) OR "FitBit"[Title/Abstract]) OR "Garmin"[Title/Abstract]) OR "Apple Watch"[Title/Abstract]) OR "ActivPAL"[Title/Abstract]) OR "Axivity"[Title/Abstract]) OR "GeneActiv"[Title/Abstract])) AND (((((((("Cohort Studies"[MeSH Terms:noexp] OR "Observation"[MeSH Terms]) OR "Observational Study"[Publication Type]) OR "Observational Studies as Topic"[MeSH Terms]) OR "cohort*"[Title/Abstract]) OR "surveillance"[Title/Abstract]) OR "cross-sectional"[Title/Abstract]) OR "observation*"[Title/Abstract]) OR "Survey"[Title/Abstract])) NOT (((("Animals"[MeSH Terms] NOT "Humans"[MeSH Terms]) AND "Randomized Controlled Trial"[Title/Abstract]) OR "RCT"[Title/Abstract]) OR "intervention"[Title/Abstract])

Web of Science

(TI=(“Sedentary Behavior” OR “Physical Activit*” OR “Physical Behav*” OR “Exerci*” OR “sedentary” OR “steps”) OR AB=(“Sedentary Behavior” OR “Physical Activit*” OR “Physical Behav*” OR “Exerci*” OR “sedentary” OR “steps”))

AND

(TI= (“Acceleromet*” OR “Fitness Track*” OR “Activity monitor”& OR “motion sensor*” OR “device-based” OR “ActiGraph” OR “Actical” OR “sensewear” OR “Actiband” OR “Actiwatch” OR “FitBit” OR “Garmin” OR “Apple Watch” OR “ActivPal” OR “Axivity” OR “GeneActiv”) OR AB = (“Acceleromet*” OR “Fitness Track*” OR “Activity monitor”& OR “motion sensor*” OR “device-based” OR “ActiGraph” OR “Actical” OR “sensewear” OR “Actiband” OR “Actiwatch” OR “FitBit” OR “Garmin” OR “Apple Watch” OR “ActivPal” OR “Axivity” OR “GeneActiv”))

AND

(TI = (“Cohort*” OR “Observ*” OR “surveil*” OR “survey” OR “cross-sectional”) OR AB = (“Cohort*” OR “Observ*” OR “surveil*” OR “survey” OR “cross-sectional”))

NOT

(TI= (“Randomized Controlled Trial” OR “RCT” OR “intervention” OR “child*”) OR AB = (“Randomized Controlled Trial” OR “RCT” OR “intervention”))

(TI=(“Sedentary Behavior” OR “Physical Activit*” OR “Physical Behav*” OR “Exerci*” OR “sedentary” OR “steps”) OR AB=(“Sedentary Behavior” OR “Physical Activit*” OR “Physical Behav*” OR “Exerci*” OR “sedentary” OR “steps”)) AND (TI= (“Acceleromet*” OR “Fitness Track*” OR “Activity monitor”& OR “motion sensor*” OR “device-based” OR “ActiGraph” OR “Actical” OR “sensewear” OR “Actiband” OR “Actiwatch” OR “Fit Bit” OR “Garmin” OR “Apple Watch” OR “ActivPal” OR “Axivity” OR “GeneActiv”) OR AB = (“Acceleromet*” OR “Fitness Track*” OR “Activity monitor”& OR “motion sensor*” OR “device-based” OR “ActiGraph” OR “Actical” OR “sensewear” OR “Actiband” OR “Actiwatch” OR “Fit Bit” OR “Garmin” OR “Apple Watch” OR “ActivPal” OR “Axivity” OR “GeneActiv”)) AND (TI = (“Cohort*” OR “Observ*” OR “surveil*” OR “survey” OR “cross-sectional”) OR AB = (“Cohort*” OR “Observ*” OR “surveil*” OR “survey” OR “cross-sectional”)) NOT (TI= (“Randomized Controlled Trial” OR “RCT” OR “intervention” OR “child*”) OR AB = (“Randomized Controlled Trial” OR “RCT” OR “intervention”))

Total – 3,821 06/01/2021

SPORTDiscus

(DE “EXERCISE” OR "PHYSICAL activity") OR SU(“EXERCISE” OR "PHYSICAL activity") OR (AB(“physical activit*” OR “physical behav*” OR “exercise” OR “sedentary” OR “steps”)) OR (TI(“physical activit*” OR “physical behav*” OR “exercise” OR “sedentary” OR “steps”))

AND

(DE " Accelerometry" OR " Fitness Trackers") OR SU(“Accelerometry” OR " Fitness Trackers") OR (AB(“acceleromet*” OR “fitness track*” OR “activity monitor*” OR “motion sensor*” OR “device-based” OR “ActiGraph” OR “Actical” OR “Sensewear” OR “Actiband” OR “Actiwatch” OR “FitBit” or “Garmin” OR “Apple Watch” OR “ActivPAL” OR “Axivity” OR “GeneActiv”)) OR (TI(“acceleromet*” OR “fitness track*” OR “activity monitor*” OR “motion sensor*” OR “device-based” OR “ActiGraph” OR “Actical” OR “Sensewear” OR “Actiband” OR “Actiwatch” OR “FitBit” or “Garmin” OR “Apple Watch” OR “ActivPAL” OR “Axivity” OR “GeneActiv”))

AND

(DE "Cohort Studies" OR " Observation") OR SU("Cohort Studies" OR " Observation") OR (AB(“cohort*” OR “surveillance” OR “cross-sectional” OR “observation*” OR “survey”)) OR (TI(“cohort*” OR “surveillance” OR “cross-sectional” OR “observation*” OR “survey”))

NOT

(AB(“Randomized Controlled Trial” OR “RCT” OR “intervention” OR “child*”)) OR (TI(“Randomized Controlled Trial” OR “RCT” OR “intervention” OR “child*”))

Total- 892 06/01/2021
