# Supplementary material for: Historical development of accelerometry measures and methods for physical activity and sedentary behavior research worldwide: A scoping review of observational studies of adults
Source: PLoS One. 2022 Nov 21;17(11):e0276890. doi: 10.1371/journal.pone.0276890 (PMC9678297; doi:10.1371/journal.pone.0276890)
Supplement: S3 File — (PDF) [file pone.0276890.s003.pdf]

Supplement 3. Checklist of reporting elements from Montoye et al. [13]

(1) Brand of accelerometer used?
Examples: Actiband, Actibelt, Actical, ActiGraph, etc.

(2) Model of accelerometer used?
Examples: Charge, GT3X+

(3) Epoch length used?
Examples: raw, 1 second, 5 seconds, 15 seconds, etc.

(4) Placement of accelerometer (location and side of the body)?
Examples: Location (ankle, wrist, etc.) and Side (dominant, left, right, etc.)

(5) Number of accelerometers distributed (number of participants enrolled at study start receiving accelerometers)?
Examples: a

(6) How were accelerometers distributed?
How were accelerometers returned? *(question added by our team)*
Examples: 1, 2, 3, etc.

(7) Days of data collection?
Examples: 1, 2, 3, etc.

(8) Criteria for defining non-wear of accelerometer?
Examples: 10 minutes of consecutive zeros, Choi et al. algorithm, etc.

(9) Number of adherent days for accelerometer data needed?
Examples: 1, 2, 3 etc.

(10) How many minutes of accelerometer data needed to be considered an adherent day?
Examples: 360 minutes, 420 minutes, etc.

(11) How was meaning derived from accelerometer data?
Examples: activity energy expenditure, activity index, average counts/epoch, average vector magnitude/epoch, cutpoints for counts/epoch, cutpoints for vector magnitude/epoch, Euclidean norm minus one, machine learning algorithms, mean amplitude deviation, sit-to-upright transitions, sitting time, standing time, steps/day*, total counts/day*, total vector magnitude/day, etc.

*Could include another time period such as an hour or week.

(12) Reported the number of people not meeting wear-time criteria, monitor malfunction, etc.
Examples: no, yes, other
