# Supplementary material for: Historical development of accelerometry measures and methods for physical activity and sedentary behavior research worldwide: A scoping review of observational studies of adults
Source: PLoS One. 2022 Nov 21;17(11):e0276890. doi: 10.1371/journal.pone.0276890 (PMC9678297; doi:10.1371/journal.pone.0276890)
Supplement: S4 File — (PDF) [file pone.0276890.s004.pdf]

Supplement 4. Location of observational studies of adults collecting accelerometry (n=155).

	Overall	
Description	%	n
Country:		
Australia	1.9	3
Belgium	0.6	1
Brazil	3.9	6
Canada	3.2	5
China	1.9	3
Denmark	2.6	4
Finland	5.2	8
France	0.6	1
Germany	1.9	3
Greenland	0.6	1
Hong Kong	0.6	1
Hungary	0.6	1
Iceland	0.6	1
Japan	10.3	16
Kenya	0.6	1
Mexico	0.6	1
Netherlands	4.5	7
New Zealand	0.6	1
Norway	2.6	4
Poland	0.6	1
Portugal	1.3	2
Singapore	0.6	1
South Korea	1.3	2
Spain	3.2	5
Sweden	3.9	6
Switzerland	1.9	3
Tasmania	0.6	1
United Kingdom*	10.3	9
United States	27.7	43
Multiple countries	4.5	7

*United Kingdom included England, Scotland, Wales, and northern Ireland
