# Supplementary material for: Historical development of accelerometry measures and methods for physical activity and sedentary behavior research worldwide: A scoping review of observational studies of adults
Source: PLoS One. 2022 Nov 21;17(11):e0276890. doi: 10.1371/journal.pone.0276890 (PMC9678297; doi:10.1371/journal.pone.0276890)
Supplement: S5 File — (PDF) [file pone.0276890.s005.pdf]

Supplement 5. Criteria for defining nonwear among accelerometers and combined method of distribution and return used by large observational studies of adults (n=166)

Description	Overall	
	%	n
Criteria for defining nonwear:		
10 minutes of consecutive zeros	0.6	1
15 minutes of non-wear	0.6	1
20 minutes of consecutive zeros	3.0	5
30 minutes of consecutive zeros	1.8	3
35 minutes when data was not recorded	0.6	1
60 minutes of consecutive zeros	10.2	17
60 minutes with consecutive <100 cpm	0.6	1
60 minutes of consecutive zeros with 1 min interruption	0.6	1
60 minutes of consecutive zeros with 2 min interruption (0-100 cpm); Troiano et al. 2008	9.6	16
60 minutes of consecutive zeros with 2 min interruption (0-200 cpm)	1.8	3
60 minutes of consecutive zeros with 2 min interruption allowed	1.2	2
60 minutes of consecutive zeros with 2 min of activity	0.6	1
60 minutes of no activity allowing for 2 min activity	1.2	2
90 minutes of consecutive zeros	1.2	2
90 minutes of consecutive zeros with 2 min interruption (0-100 cpm)	0.6	1
90 minutes of zero steps	0.6	1
100 minutes of consecutive zeros	0.6	1
120 minutes of zero steps	0.6	1
150 minutes of consecutive zeros	0.6	1
180 minutes of consecutive zeros	0.6	1
240 minutes with no movement	0.6	1
600 minutes of consecutive zeros	0.6	1
Choi algorithm (60 min consecutive zeros, 10 to 60 min window, 2 min interruption)	0.6	1
Choi algorithm (60 min consecutive zeros, 30 min window, 2 min interruption)	0.6	1
Choi algorithm (90 min consecutive zeros)	1.2	2
Choi algorithm (90 min consecutive zeros, 30 min window, 2 min interruption); Choi et al. 2011	6.6	11
Choi algorithm (90 min consecutive zeros, 90 min window, 2 min interruption)	0.6	1
Sleep algorithm and Choi algorithm (90 min consecutive zeros, 30 min window)	0.6	1
Other using sitting, standing, and steps	1.8	3
Manufacturer algorithm	0.6	1
Sensewear proprietary algorithm	0.6	1
Based on raw data	12.0	20
Based on capacitive sensor	0.6	1
Based on galvanic heat sensor	0.6	1
Loss of skin contact	0.6	1
Days with greater than 5% of data missing were excluded	0.6	1
Detected by the device	0.6	1
Heart rate and inactivity	0.6	1
Logbook	1.2	2
Single use attachment with researcher removing it	0.6	1
Acceleration from the 3 axes remained within 187.5 mG range for >=30 min	0.6	1
Not indicated	30.1	50

Combined method of data collection (distribute/return):		
In-person / in-person	13.9	23
Mail / mail	11.5	19
In-person / mail	16.3	27
In-person / not indicated	13.3	22
Mail / not indicated	4.2	7
Not indicated / in-person	1.8	3
Not indicated / mail	1.2	2
Not applicable (distributed cohort)	3.0	5
Other	3.6	6
Not indicated / not indicated	31.3	52

Abbreviations: mG milli G force; min, minutes

Note: Eight studies used 2 accelerometers, and one study used 4 accelerometers; therefore, the sample size was n=166 for this table. We selected a sample of studies and n=29 responded to check their entries and fill in missing information when possible. Therefore, the "not indicated" category is reduced when the study provided missing information from the selected study.

References:

Troiano, R., D. Berrigan, K. Dodd, L. Masse, T. Tilert and M. McDowell (2008). "Physical activity in the United States measured by accelerometer." Med Sci Sports Exerc 40(1): 181-188.

Choi, L., Z. Liu, C. E. Matthews and M. S. Buchowski (2011). "Validation of accelerometer wear and nonwear time classification algorithm." Med Sci Sports Exerc 43(2): 357-364.
